# Disease entity impacts muscle wasting in the ICU with COVID-19 patients losing muscle nearly twice as fast

**DOI:** 10.1038/s41598-025-05912-2

**Published:** 2025-06-20

**Authors:** Johannes Kolck, Clarissa Hosse, Uli Fehrenbach, Nick L. Beetz, Timo A. Auer, Christian Pille, Dominik Geisel

**Affiliations:** 1 Department of Radiology, Charité, Berlin, Germany; 2 Berlin Institute of Health at Charité, Berlin, Germany; 3 Department of Anesthesiology and Intensive Care Medicine | CCM | CVK, Charité, Berlin, Germany

**Keywords:** Critical care, Acute pancreatitis, COVID-19, Muscle wasting, Artificial intelligence, Computed tomography, Health care, Medical research

## Abstract

Muscle loss in critically ill patients, particularly during prolonged ICU stays, poses significant challenges to recovery and long-term outcomes. ICU-acquired weakness (ICUAW) manifests as severe muscle depletion, correlating with illness severity and hospitalization duration. This study aims to characterize long-term muscle loss trajectories in ICU patients with acute respiratory distress syndrome (ARDS) due to COVID-19 and severe acute pancreatitis (AP) and to explore contributing factors to elevated muscle decay. Retrospective cohort study including 154 ICU patients, 100 individuals suffering from AP and 54 from COVID-19 ARDS, who underwent a minimum of three CT scans during hospitalization, totaling 988 assessments. Sequential segmentation of psoas muscle area (PMA) was performed, and relative muscle loss per day for the entire monitoring period, as well as for the interval between each consecutive scan, was calculated. Bivariate and multivariate linear regression analyses were conducted to identify and evaluate the factors contributing to muscle loss. ICU patients experienced an average PMA decline of 46.0%, with a reduction of 41.8% observed in COVID-19 patients and 48.2% in AP patients. Notably, the long-term daily PMA loss was significantly greater in COVID-19 patients (1.88%) compared to AP patients (0.98%; *p* < 0.001). Linear regression analysis identified disease entity (*p* < 0.001), length of hospitalization (*p* < 0.001), and obesity as significant contributors to daily muscle deterioration. Patients admitted to the ICU for COVID-19 and severe AP can experience extreme muscle decay, reaching up to 48.2%. While decay rates vary considerably, COVID-19 patients experienced nearly twice the daily muscle loss compared to AP patients. Key factors contributing to muscle decay included disease entity, hospitalization duration, and obesity. These findings highlight the distinct impact of the underlying disease on muscle deterioration and emphasize the heightened risk for obese patients and those undergoing extended hospitalization.

## Introduction

Muscle loss in critically ill patients, particularly during prolonged hospitalization, poses significant challenges to their recovery and long-term outcomes. Intensive care unit (ICU)-acquired weakness (ICUAW), a common complication in such patients, manifests as severe depletion of muscle mass and function^1–3^. The onset of muscle wasting typically occurs shortly after admission to the ICU and worsens progressively over time^4,5^. The degree of muscle loss correlates with the severity of the underlying illness and the duration of hospitalization, with notably pronounced effects observed in patients diagnosed with sepsis^6^.

While several studies have documented muscle loss in the initial days following ICU admission, as highlighted in a recent meta-analysis encompassing 3251 critically ill patients^7^, comprehensive understanding of long-term muscle decay remains limited, with only a few studies addressing this aspect, typically involving small and heterogeneous cohorts^8–12^.

To address these gaps, we have previously used artificial intelligence (AI)-based muscle monitoring by segmenting clinically indicated computed tomography (CT) scans to analyze muscle wasting in two homogeneous cohorts. The first cohort included patients with severe SARS-CoV-2 infection, characterized by high mortality and significant muscle wasting^11^. Similarly, the second cohort included patients with severe pancreatitis, a condition known to be associated with significant mortality, prolonged ICU stays and profound physiological deterioration^13–15^. By integrating muscle wasting data from both cohorts, this study aimed to provide a comprehensive perspective on long-term muscle wasting trajectories. In addition, we sought to identify the key factors influencing the dynamics of muscle wasting in these distinct patient populations.

## Materials and methods

### Ethics approval

The study received approval from the Institutional Review Board (Internal registration number: EA4/152/20) and adhered to the principles outlined in the Declaration of Helsinki. Due to the retrospective nature of the study, the Institutional Review Board of Charité waived the need of obtaining informed consent.

### Study design and patient population

In this study, we conducted a retrospective analysis of changes in psoas muscle area (PMA) among patients admitted to the ICU due to either SARS-Cov-2 virus infection or AP. For patient selection, we screened our institutional database for individuals admitted with acute pancreatitis (AP) between January 2012 and December 2022, and with SARS-CoV-2 infection between March 2020 and January 2022. Inclusion criteria for both groups included adult age, an intensive care unit (ICU) stay of at least 10 days, and the availability of a minimum of three abdominal CT scans obtained during hospitalization, with the first scan performed within the first week of or prior to ICU admission. The following covariates were retrospectively collected: age, gender, body mass index (BMI), overweight, obesity, sarcopenia, and preconditions at ICU admission. In contrast, hospitalization duration (days), ICU stay (days), days of invasive mechanical ventilation (IMV), Sequential Organ Failure Assessment (SOFA) score at ICU admission, and the highest SOFA score were recorded based on the full course of hospitalization.

### Segmentation of tissue compartments

Patient tissue compartment quantification was conducted using an AI-based automated image segmentation tool integrated into the hospital’s Picture Archiving and Communication System (PACS) software (Visage version 7.1., Visage Imaging GmbH, Berlin, Germany), previously validated in other studies^11,16^. Following automated identification of the third lumbar vertebra (L3) level, the system performed segmentation to distinguish tissues into subcutaneous fat (SAT), skeletal muscle area (SMA), visceral fat (VAT), and psoas muscle area (PMA). The software then calculated the areas in square centimeters (cm^2^) for each component. These values are not automatically standardized to body surface area. Manual corrections were made by an experienced radiologist (JK) after reviewing each automated segmentation, if deemed necessary.

### Definitions

Overweight and obesity were defined following internationally recognised BMI thresholds: BMI > 25 kg/m^2^ for overweight and BMI > 30 kg/m^2^ for obesity.

Sarcopenia was determined using gender-specific cut-offs: SMA < 34.3 cm^2^ for women, and < 45.4 cm^2^ for men, based on established literature^17^. Sarcopenia was assessed at a single time point, specifically at the scan closest to ICU admission, based on the muscle cross-sectional area at the L3 vertebral level.

### Muscle decay rates

To simplify the analysis of muscle decay, we defined four key metrics: initial loss rate, maximum loss rate, long-term loss rate, and total loss. All muscle loss rates were calculated by subtracting the respective psoas muscle areas (in cm^2^) between two CT scans and dividing the difference by the number of days between them.

- Initial loss rate was calculated between the first and second CT scans.
- Maximum loss rate was determined by performing this calculation across all consecutive scan pairs, with the highest rate selected as the maximum.
- Long-term loss rate was calculated between the first and last CT scans.

The total loss represents the relative reduction in muscle area between the first (baseline) and the last CT scan, independent of the time interval—e.g., a 35% loss at the final time point compared to baseline.

### Statistics

Descriptive statistics are presented as means and standard deviations. The Mann–Whitney U test was used to compare muscle loss rates and clinical variables between entity groups. A linear mixed model analysis was used to generate a figure comparing the absolute long-term loss of both groups. Linear regression analyses were applied to assess the relationship of daily muscle decay rates with potentially contributing factors. All *p*-values less than 0.05 were considered statistically significant. Statistical analyses were performed utilizing Jamovi Version 2.3 (The jamovi project, Sydney, Australia).

## Results

### Demographic data

#### Acute pancreatitis group

**Fig. 1:**
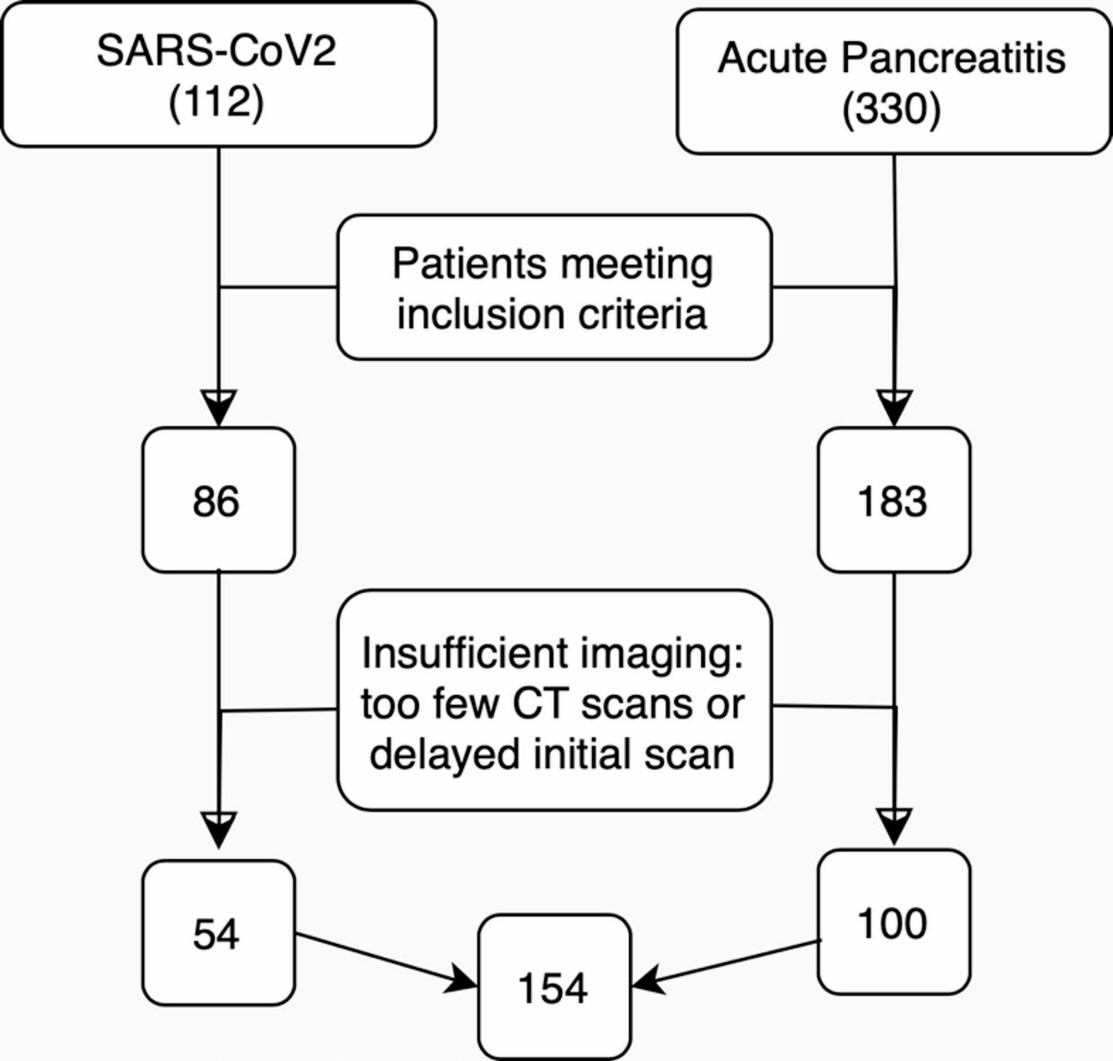
Flow chart of patient enrollment for both cohorts.

One hundred patients, 75 men and 25 women, admitted for the ICU due to AP met the inclusion criteria. The mean age of the study population upon hospital admission was 60.17 years, ranging from 19 to 94 years. The mean Body Mass Index (BMI) was 26.3 kg/m^2^, ranging from 16.67 to 52.59 kg/m^2^. Sarcopenia was present in 42% of patients upon admission, while 55% were classified as obese with a BMI > 25 kg/m^2^., and 19% were considered severely obese with a BMI > 30 kg/m^2^. The patient enrolment flowchart is shown in Fig. 1.

### SARS-CoV-2 group

A total of 54 critically ill, 38 men and 16 women, with severe ARDS due to SARS-CoV-2 infection were enrolled. The mean age of the study population upon hospital admission was 55.74 years, ranging from 28 to 79 years. The mean BMI was 29.74, ranging from 17.18 to 51.78. Upon admission, sarcopenia was observed in 35% of patients, with 61% classified as obese (BMI > 25 kg/m^2^) and 26% as severely obese (BMI > 30 kg/m^2^). Average BMI was significantly higher in the SARS-CoV-2 group compared to patients with AP (*p* < 0.001).

### Preconditions and hospitalization

The final study cohort included 154 patients who were originally part of previous studies^11,14^, yielding a total of 988 CT scans. The broad majority of enrolled patients was diagnosed with chronic preconditions (73.4%). The most prevalent condition was arterial hypertension (74/154 patients), followed by other cardio vascular diseases (44/154 patients), diabetes (28/154) and pulmonal (22/154) preconditions. AP patients had significantly more preconditions compared to the COVID-19 group (*p* < 0.001). Mean hospital length of stay of the whole collective was 103.87 days, with 76.35 days at the ICU. Patient with AP had a significantly longer total hospitalization time with on average 116.63 days vs. 80.24 in patients with SARS-CoV-2 infection (*p* = 0.004). Time spent at the ICU was shorter among SARS-CoV-2 patients (65.17 vs. 82.39 days; *p* = 0.085). Mean SOFA score at ICU admittance was 9.7 ± 4.5. Initial SOFA was significantly higher in the COVID-19 group 11.9 vs. 8.4 in AP patients (*p* = 0.002), whereas maximal SOFA scores and survival rates of 56% and 59% did not differ significantly. Results are compiled in Table 1.

**Table 1:** Overview of patient collective, divided into disease entity groups: SARS-CoV-2 (COVID-19) and acute pancreatitis (AP).

	COVID-19	AP	Total	*p*-value
Age	55.7 ± 12.2	60.2 ± 17.0	59.0 ± 15.6	**0.09**
Female gender	29.60%	26.00%	27.30%	0.629
BMI	29.7 ± 6.76	26.3 ± 5.49	27.5 ± 5.9	** < 0.001**
Preconditions	50.00%	86.00%	73.40%	** < 0.001**
Hospitalization (days)	80.2 ± 50.7	116.6 ± 82.7	103.9 ± 75.0	**0.007**
ICU stay (days)	65.2 ± 46.1	82.4 ± 64.7	76.4 ± 58.2	0.107
Days of IMV	56.0 ± 41.2	53.8 ± 47.6	54.6 ± 45.4	0.426
SOFA at ICU admission	11.9 ± 3.9	8.4 ± 5.4	9.7 ± 4.8	**0.002**
Highest SOFA	13.9 ± 3.6	13.0 ± 4.4	13.3 ± 4.1	0.511
Overweight	61.10%	55.20%	57.30%	0.817
Obesity	25.90%	19.30%	21.60%	0.783
Sarcopenia	35.20%	42.00%	39.00%	0.48
Survival	56.60%	59.00%	57.80%	0.68
Avg. muscle loss/day	1.88% ± 1.62%	0.98% ± 0.81%	1.28% ± 1.21%	** < 0.001**
Total Muscle loss	41.8% ± 22.4°% 48.2% ± 20.7% 46.0% ± 21.3%	0.229		

Significant differences between the groups are printed in bold.

### Muscle loss during hospitalization

**Fig. 2:**
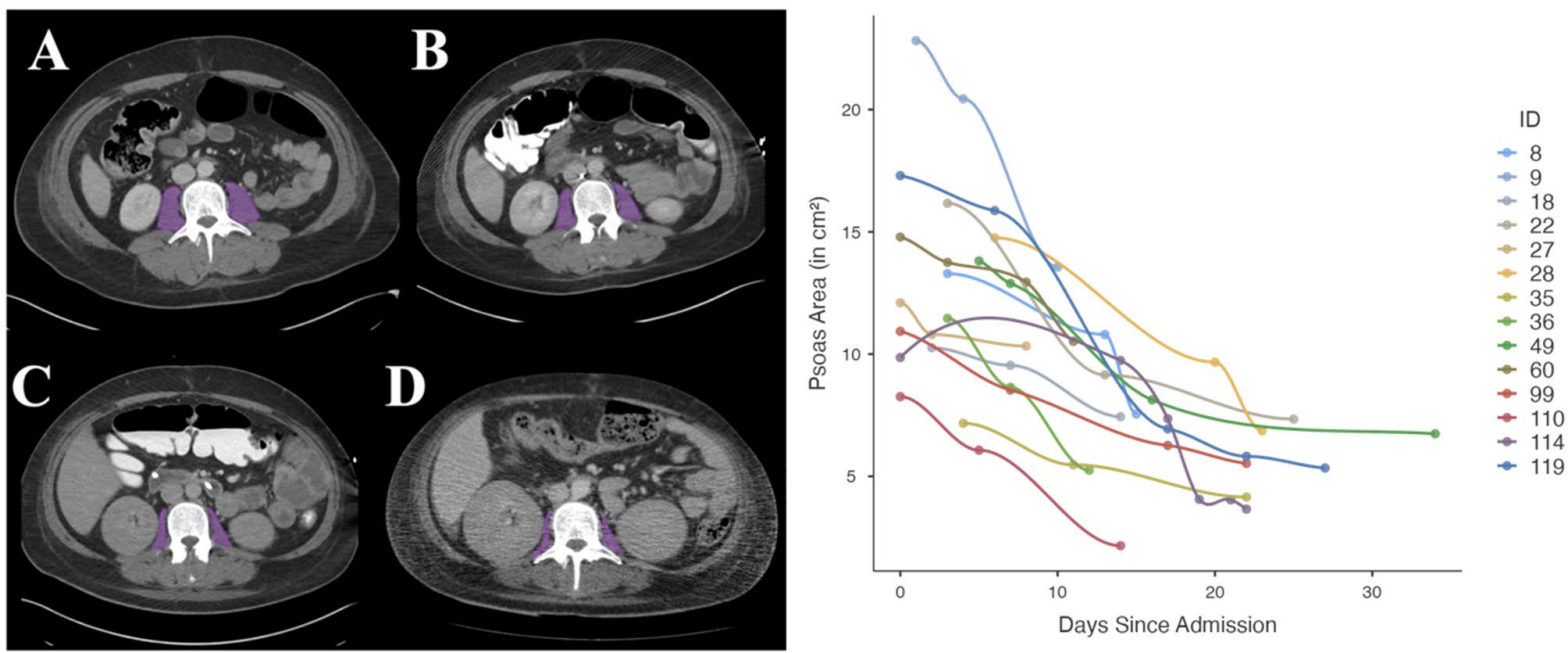
Left: PMA assessments based on clinically indicated CT scans of a COVID-19 Patient during hospitalization. (**A**) First CT scan at ICU admission, followed by scans after 7 days (**B**), 25 days (**C**) and 57 days (**D**). Right: Illustration of muscle loss in selected IDs measured using clinically indicated CT scans.

**Fig. 3:**
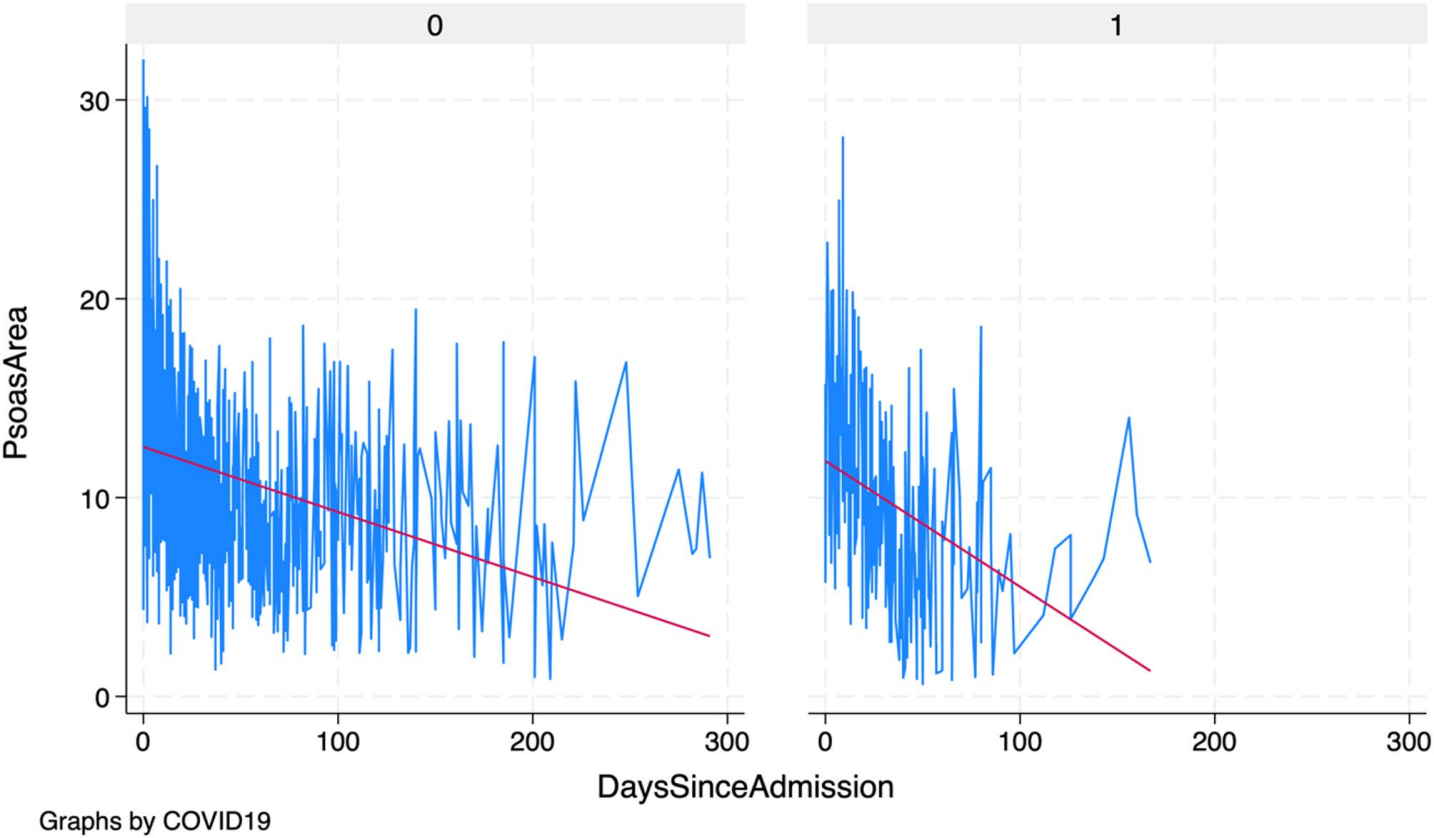
Fitted values of linear mixed model analysis comparing absolute PMA measurements, grouped by entity (0 = acute pancreatitis; 1 = COVID-19). The graph shows the significantly faster muscle loss in the COVID-19 group.

In both groups, the psoas muscle served as the reference muscle area. The observed cumulative muscle loss exhibited a nonlinear pattern, characterized by an overall negative trend with varying rates of decline at different time intervals. On average, patients experienced a total psoas muscle area (PMA) loss of 46.0%, with 41.8% in COVID-19 patients and 48.2% in acute pancreatitis (AP) patients. The initial loss rate was 2.33% per day among AP patients and 2.82% among COVID-19 patients. Long-term PMA loss per day diverged significantly between patients admitted for ARDS due to SARS-CoV-2 infection with a mean loss rate of 1.88% per day, while patients with AP exhibited a loss of 0.98% per day (*p* < 0.001; Table 1 and Fig. 2 and 3).

### Contributors to average daily muscle loss

All available variables were analyzed for their correlation with the average rate of muscle loss per day using a bivariate analysis. Statistically significant factors identified included disease entity (*p* < 0.001), BMI (*p* = 0.004), the presence of pre-existing conditions (*p* = 0.005), length of hospitalization (*p* < 0.001), ICU stay duration (*p* < 0.001), days of invasive mechanical ventilation (IMV, *p* < 0.001), initial SOFA score (*p* = 0.044) and the presence of obesity (*p* = 0.032). In contrast, variables such as initial total muscle area, psoas muscle area, PMI, SMI, VAT, SAT, patient age, gender, arterial hypertension, other cardiovascular conditions, pulmonary or oncological conditions, and the presence of sarcopenia, sarcopenic obesity and maximum SOFA scores did not reach statistical significance. In the multivariable linear regression model, only three variables remained significant: disease entity (*p* = 0.012), length of hospitalization (*p* = 0.005), and obesity (*p* = 0.012). A detailed summary of these findings is presented in Tables 2 and 3.

**Table 2:** Bivariate linear regression of average muscle loss per day.

Predictor	Estimate	SE	Lower CI	Upper CI	*p*
Age	1.79E−05	6.67E−05	− 1.13E−04	1.49E−04	0.789
Female gender	6.33E−04	2.23E−03	− 3.74E−03	5.00E−03	0.776
BMI (kg/m^2^)	4.58E−04	1.56E−04	1.52E−04	7.64E−04	**0.004**
Psoas Area (in cm^2^)	1.20E−04	1.68E−04	− 2.09E−04	4.49E−04	0.476
SATArea (in cm^2^)	− 5.57E−06	7.55E−06	− 2.04E−05	9.23E−06	0.462
VATArea (in cm^2^)	4.89E−06	9.49E− 06	− 1.37E−05	2.35E−05	0.607
Disease entity	8.90E−03	1.99E−03	5.00E−03	1.28E−02	**0.001**
Preconditions	6.38E−03	2.24E−03	1.99E−03	1.08E−02	**0.005**
Art. hypertention	3.67E−03	1.97E−03	− 1.91E−04	7.53E−03	0.064
Diabetes	2.76E−03	2.54E−03	− 2.22E−03	7.74E−03	0.278
Cardio-vascular	2.24E−03	2.19E−03	− 2.05E−03	6.53E−03	0.307
Pulmonal	− 7.56E−04	2.86E−03	− 6.36E−03	4.85E−03	0.792
Malignant	4.02E−03	2.84E−03	− 1.55E−03	9.59E−03	0.159
Hospitalization (days)	7.32E−05	1.17E−05	5.03E−05	9.61E−05	**0.001**
ICU stay (days)	8.05E−05	1.54E−05	5.03E−05	1.11E−04	**0.001**
Days of IMV	8.03E−05	2.13E−05	3.86E−05	1.22E−04	**0.001**
Highest SOFA	8.54E−04	5.12E−04	− 1.50E−04	1.86E−03	0.101
Overweight	3.53E−03	2.03E−03	− 4.49E−04	7.51E−03	0.084
Obesity	4.80E−03	2.21E−03	4.68E−04	9.13E−03	**0.032**
Sarcopenia	− 9.19E−05	9.19E−05	− 2.72E−04	8.82E−05	0.319
Sarcopenic obesity	3.22E−03	2.57E−03	− 1.82E−03	8.26E−03	0.211

Significant variables are printed in bold. BMI, Body Mass Index, SAT, Subcutaneous Adipose Tissue, VAT, Visceral Adipose Tissue, ICU, Intensive Care Unit, IMV, Invasive Mechanical Ventilation, SOFA, Sequential Organ Failure Assessment. CI, Confidence interval.

**Table 3:** Multivariate linear regression of muscle loss per day.

Predictor	Estimate	SE	Lower CI	Upper CI	*p*
Disease	5.21E−03	2.04E−03	1.21E−03	9.21E−03	**0.012**
BMI (kg/m^2^)	2.66E−04	1.41E−04	− 1.04E−05	5.42E−04	0.061
Preconditions	2.32E−03	2.10E−03	− 1.80E−03	6.44E−03	0.271
Hospitalization (days)	6.17E−05	2.15E−05	1.96E−05	1.04E−04	**0.005**
ICU stay (days)	− 1.04E−05	2.93E−05	− 6.78E−05	4.70E−05	0.723
Days of IMV	2.77E−05	2.36E−05	− 1.86E−05	7.40E−05	0.244
Obesity	4.85E−03	1.91E−03	1.11E−03	8.59E−03	**0.012**

Significant variables are printed in bold. CI = Confidence interval.

## Discussion

This study analyses the long-term muscle decay from two severely ill ICU cohorts: 54 patients with ARDS due to COVID-19 pneumonia and 100 patients with severe acute pancreatitis (AP), representing the largest cohort in this field to date. Both groups showed substantial muscle loss, with an average total PMA decline of 46.0% (41.8% in COVID-19 and 48.2% in AP patients). Notably, the average muscle loss per day was nearly twice as high in COVID-19 patients compared to AP patients (1.88% vs. 0.98%; *p* < 0.001; Mann–Whitney U test). Linear regression identified longer hospital stays (*p* = 0.005), obesity (*p* = 0.012), and disease type (*p* = 0.012) as significant contributors to increased muscle deterioration.

Our study differs from previous research in several key aspects. First, our dataset covers longer hospitalization periods, unlike most prior studies that focus only on the initial days following ICU admission^7^. Second, all patients in this study experienced prolonged ICU stays exceeding ten days^18^, providing valuable insights into muscle depletion during later phases of critical illness. Thirdly, we are the first to compare muscle wasting rates across different disease entities, demonstrating the significant impact of disease type on the progression of muscle wasting.

In reviewing the existing literature, our findings confirm several well-established contributors to muscle wasting. ICU patients frequently experience rapid muscle wasting due to a combination of factors. Immobility leads to reduced mechanical loading, particularly on type I muscle fibers, leading to reduced protein synthesis and increased catabolism^19^. Systemic inflammation induces catabolic pathways via cytokines and mitochondrial dysfunction^20^. Neuromuscular dysfunction, including Critical illness polyneuropathy (CIP) and myopathy (CIM), affects nerve and muscle excitability^21^. Hormonal imbalances, up-regulation of cortisol and down-regulation of Insulin/IGF-1, as well as insulin resistance promote catabolism and energy depletion^22^. Finally, nutritional deficiencies limit the amino acids needed for muscle repair, exacerbating muscle loss^23^. Among these immobilization, emerges as a key factor, with its effects increasing with prolonged exposure^24^ and potentially exacerbated by the use of neuromuscular blocking agents (NMBAs) during IMV^25,26^. The lack of significance for IMV duration in our analysis is likely due to its consistent application in both cohorts. A similar pattern was observed for age and comorbidities, which did not differ between groups in our study, in contrast to findings from previous studies^27,28^.

The impact of obesity on muscle wasting in intensive care patients is still debated. Previous research showed that critically ill obese patients may experience muscle loss differently from non-obese individuals, showing better muscle quality on admission and a slower decline in the first 4–5 days compared with non-obese patients. However, the same study showed that despite the initial advantage, obese patients still experienced significant muscle loss over time^29^. While obesity is associated with certain protective effects, it also introduces challenges^30^, particularly in the conditions investigated here. In COVID-19 patients an elevated BMI has been linked to increased inflammatory responses, as well as higher mortality and morbidity rates^31–33^. Likewise, higher BMI has been associated with more complications and poorer outcomes in AP^34,35^. Consequently, the effect of obesity on increased muscle wasting observed in this study may be specific to, or more pronounced in, these two conditions.

The comparison of muscle wasting rates between disease entities provides new insights that add to the existing body of knowledge. In particular, both disease entities had elevated initial muscle loss rates, with AP patients experiencing a loss of 2.33% per day and COVID-19 patients experiencing a loss of 2.82% per day. These rates surpass the average of 2% daily muscle loss during the first week of ICU admission reported in the above-mentioned meta-analysis during the first week of ICU admission^7^. However, neither the initial muscle loss rate nor the total muscle decay between admission and discharge or death, 41.8% in COVID-19 and 48.2% in AP patients, diverged significantly between the two entity groups. In the AP group, the muscle wasting rate dropped from a high level at the early course to just below 1% averaged over the total stay. In contrast, in the COVID-19 patients, loss rates remained at a high level, resulting in an average daily loss rate significantly higher, almost twice as high, at 1.88% (*p* < 0.001). This is even more remarkable, as the AP group had significantly longer hospital stays and showed more comorbidities at admittance, both known risk factors for elevated muscle decay^27^. While the underlying cause for the different rates of muscle wasting between disease entities remain obscure, it may be hypothesized that they are likely to be due to the severity of the disease, which posed unprecedented challenges to health systems in many countries during the COVID-19 pandemic^36^. The rate of muscle decay can thus be seen as a biomarker for disease severity, which may help to evaluate patients additionally to already implemented scoring systems like SOFA. In contrast to disease severity scores, which are designed to asses patient’s acute status, muscle decay patterns may be used to quantitatively describe disease impact in relation patient’s status at admission*,* thus potentially serving as a valuable asset for informed decision-making.

### Limitations

Given the retrospective nature of the study, some degree of selection bias is inevitable. This is likely to lead to under-representation of less severely ill patients, as the inclusion criteria—such as the requirement for three available CT scans—may favor those with more complex clinical courses. In addition, our study did not consider several potential confounders, such as variations in nutritional interventions or the use of medications such as corticosteroids, which are known to affect muscle loss and recovery in critically ill patients. As our study relied on clinically indicated CT scans rather than pre-determined intervals, making it difficult to determine the exact loss on each day of hospitalization. Moreover, even though both the length of stay in the ICU and the duration of invasive mechanical ventilation contribute to and result in muscle wasting^37^, the results of our study do not imply a causal relationship. Furthermore, the methodology used in this study presents muscle loss as a linear process, even though it follows a more complex, non-linear trajectory. Nevertheless, this approach allowed a comprehensive long-term study of muscle wasting, a topic that has rarely been studied and never in the cohorts presented here. In addition, our method offers greater precision than alternatives such as ultrasound, as highlighted by other authors and recommended by guidelines for nutritional status assessment^38–40^. Moreover, our study benefits from a substantial cohort comprising 154 patients, the largest population investigated for long-term muscle loss in ICU patients to date^7^.

## Conclusion

Long-term muscle loss in critically ill patients with COVID-19-induced ARDS or severe AP is substantial, reaching up to 48.2%. Notably, the average daily muscle loss was nearly twice as high in COVID-19 patients. Our results highlight the significant influence of underlying disease on muscle loss, and the increased vulnerability of obese patients and those with longer hospital stays.

## Data Availability

The datasets generated and analyzed during the current study are available from the corresponding author on reasonable request.

## Abbreviations

AI: Artificial intelligence
AP: Acute pancreatitis
ARDS: Acute respiratory distress syndrome
BMI: Body mass index
CT: Computed tomography
ICU: Intensive care unit
ICUAW: ICU-acquired weakness
IMV: Invasive mechanical ventilation
NMBAs: Neuromuscular blocking agents
PCS: Picture archiving and communication system
PMI: Psoas muscle index
PMA: Psoas muscle area
SAT: Subcutaneous fat
SMA: Skeletal muscle area
SMI: Skeletal muscle index
SOFA: Sequential organ failure assessment
VAT: Visceral fat
